# Work, Identity, Place, and Population. A Changing Landscape

**DOI:** 10.3389/fsoc.2020.00068

**Published:** 2020-09-08

**Authors:** Julia Bennett

**Affiliations:** Department of Social and Political Science, University of Chester, Chester, United Kingdom

**Keywords:** biography, work, taskscape, life-history, postindustrial, narrative, place, social class

## Abstract

Taking a biographical approach, this paper uses life history narratives across four generations of families living and working in Wigan, Lancashire to analyse social and cultural changes in working life biographies over the past 80 years. Beginning with those who left school at 14, prior to the 1944 Education Act up to the present, where young people are required to remain in education until 18, the paper examines the decisions people have taken throughout their working lives. Inevitably these are shaped by structural changes, particularly to the industrial landscape. The biographical narratives allow a “bottom up” approach to uncovering changes to life courses over three generations in a northern British former industrial town whilst also exploring the wider relations between self, society and place (conceptualized here as “taskscape”) in a post-industrial setting. Key changes over the generations are the increased ability of women to pursue careers in addition to having a family, the decrease in parental influence over career choice, and the loss of a “job for life” and employment opportunities for manual workers.

## Introduction

This paper considers life history narratives of work from the 1940s to the present day. Using biographical narrative interviews with up to four generations of families from Wigan, UK, the choices, opportunities, and trajectories of working life are examined across the life course, in relation to changes in the type of work available, education, social policies, and place.

In post-industrial societies, biographies are often framed in a temporal and deterministic way that can imply rites of passage are standardized and relatively immobile (Hörschelmann, 2011). By looking at family narratives based in a single place, rather than focusing on individuals *per se*, this paper is able to tease apart some of the changes in working lives over a period of time which encompasses those starting work before the Welfare State, during the years of the provision of a state “safety net” and into the current era of neoliberal subjectivity. Continuities as well as differences over time become visible in the connections between self, society, and place and the dynamic nature of the lifecourse comes to the fore through the biographical approach (Bertaux and Delcroix, 2000; Bertaux-Wiame, 2005). Transitions studies tend to differentiate between the relative influence of structure or agency (Heinz and Krüger, 2001) but by examining linked lives over multiple generations elements of both structural change and personal choice can be drawn out. The significance of place on the lifecourse is a constant thread running through the narratives presented here (Hörschelmann, 2011).

Shildrick et al. (2015) ask whether “we need a longer-term historical perspective so as to examine social continuities with earlier periods prior to the boom decades and possibilities of social mobility found in the 1950s–70s?”. This paper will go some way toward addressing Shildrick et al. (2015) question. It shows changes over generations by examining work-life biographies from the perspective of those now retired, in mid-career, and looking to gain their first job. These narratives cover working lives pre-and post- the 1944 Education Act, through the full employment years of the mid-twentieth century and the mass unemployment of the 1980s into the current era of mass higher education. All of the participants here own their own home, or are currently living with their parents who are homeowners. They could be described as what Byrne (2019) has called the “missing middle”: those who are not the most deprived in society, most would describe themselves as comfortable. As Shildrick et al. (2015) also highlight “these are not just questions for youth studies, but for sociology in general,” focusing here on the dynamism of working lives.

In deindustrialisation discourses an emphasis on the intergenerational impact may obscure the salient issue of place. Place is a key marker of belonging and identity for people. In the activities of daily life in a familiar place, place appears to be merely a context for daily life. But place is, in fact, a part of what makes up the ordinariness of daily activity (Bennett, 2015). As Valentine (2003, p. 41) says “place matters” when it comes to the type of work available and the ability to access it through appropriate social networks. The impact of closures of large industries, or work places of any kind, goes beyond those who worked there and extends to the wider community (Walkerdine and Jimenez, 2012), particularly social and leisure activities. Part of this impact is economic but it transcends production and consumption. When the identity of a place is built around an industry its loss changes the identity of that place, which then reflects back on to the people who live there (Rhodes et al., 2019, p. 33; Bennett, 2015). As Rhodes et al. (2019) point out “place is central” when discussing deindustrialisation and changes in working biographies (see also Byrne, 2019). Therefore, rather than a focus on “work” as separate from “life,” the intention here is to show how life is made up of work, a community (in place) and a social identity—that is, in Ingold's (2000) terms, a “taskscape.” “Taskscape” describes the mutual, generally routine and often unnoticed, practices of interaction between people and environment, over time, often multiple human life times; or, as Ingold (2000, p. 195) defines it an “ensemble of tasks, performed in series or in parallel, and usually by many people working together.” Looking at life lived within a taskscape provides a focus on the processes of change, rather than only the outcomes (Bertaux and Thompson, 1996). Conceptualizing lifecourses as taskscapes allows us to see recurring rhythms across generations and the myriad interactions which go to make up the continuities and changes in lives, embedded in landscapes, over time. This paper therefore situates the changing landscape of working lives within a longitudinal, multi-generational biographical framework of both place (Bennett, 2015; Pedersen and Gram, 2018; Farrugia, 2019) and family (Bertaux and Thompson, 1996; Brannen, 2003, 2006; Nilsen and Brannen, 2014; Vogt, 2019).

I argue that being from a particular place is as fundamental as social class in how one is recognized by others and is constitutive of identities (Bennett, 2015). Moving to find work is not necessarily an appropriate choice for everyone and some value their place attachment over other life choices (Farrugia, 2019). This research is based in a single place, Wigan, in the north west of England, situated between the large cities of Manchester and Liverpool. Wigan was chosen as the site for the research because it has a high proportion of families who have “stayed put” over time (Office for National Statistics, 2019), allowing for an analysis of belonging in place through family connections. By extending the analysis to the social relations within the wider community beyond the workplace changes in employment opportunities over time become visible (Hörschelmann, 2011). Bringing together structural changes to education and the industrial landscape, the narratives here covering the years from the 1940s through to 2017, show both changes and continuities over time.

The next section discusses the methodological issues of biographical narratives and intergenerational research with families. This is followed by an explanation of the data collection, including a detailed description of the place, Wigan. The analysis itself is split into sections covering the concept of “a job for life,” deindustrialisation, changes and similarities in women's working lives and family influences on working lives.

## Biographical Narrative Research With Families

Biographical narratives are a long-established method of examining working lives sociologically (for example Thomas and Znaniecki, 1958; Foman, 1978; Bertaux, 1981). This type of interview offers researchers a unique insight into people's everyday lives. The way that a life story is narrated, the structure of the narrative, can demonstrate the importance people attach to particular events in their own lives (Kohli, 1981). This inductive methodology allows for following the research participant as they find their way along their path in life, without trying to map standardized cultural transitions onto a life story. Standard life transitions may or may not be included as important “moments” within the biography.

A life story comes into being in the telling and may be different each time it is told. Within the boundaries of “making sense” the same set of events can be organized around different plots (Czarniawska, 2004, p. 7). Biographies are rarely told in strict chronological order as the narrative follows threads and knots in different directions. Untangling these threads may be necessary to bring some order to the data, but the narrative as a unified text, knots and all, comprises the “taskscape” (Ingold, 2000, p. 195) which meshes together people (lives), place, histories, and practices. It is through this mesh that the whole story of how, why, where, when this person became who they are, becomes apparent. Enrolling multiple generations of the same family drew a picture of the family, its ethos and position, that I did not get from the individual respondents whose families did not take part. A family narrative highlights relations between family members bringing social-structural relations within the community and the place to the fore (Bertaux and Delcroix, 2000, p. 75), avoiding the individual focus biographical research can sometimes take (Hörschelmann, 2011).

This research uses families in a particular place, as the locus of the empirical work. Individual life stories gathered from people within a particular community can tell us about individual lives within that community, but extending a biographical approach to multiple members of the same family within a community begins to show how the taskscape is reproduced in that community (Bertaux and Delcroix, 2000, p. 75). A family spread across multiple households within a small area is likely to be a part of the fabric of that place community, sharing the same social connections (Nilsen and Brannen, 2014). Support networks within the family and the community as a whole create “both potential opportunities and potential constraints for its members” (Bertaux-Wiame, 2005, p. 49). Opportunities include being privy to job opportunities before these are opened up to the public whilst constraints may involve remaining in the place to look after family members (Brannen, 2003). Lives can sometimes seem to follow a similar trajectory, from one generation to the next but there are always rejections, conflicts, varying degrees of external pressure giving rise to changes in the family form (Brannen, 2003). One child may stay put but another leaves, for example. Researching families encompasses wider sociological notions than the family itself: families are connected to places through houses which may be passed on within the family; through workplaces where many family members work; through attending the same schools, even with the same teachers; and through cemeteries and memorials. These all build strong connections to the place (Farrugia, 2019).

The next section outlines the wider research project that the narratives used here are a part of.

## The Research Project

The research project took place in Wigan, England between 2009 and 2017. The first stage of the research in 2009–10 comprised two three-generation families and three two-generation families alongside five individuals whose families did not want to participate. Participants were found through local advertising and snowball techniques. Overall 22 biographical interviews were held. In addition, ten of the participants completed written and photographic diaries over the course of a week and I subsequently conducted six post-diary interviews. In 2016–17 I contacted some of the original participants to take part in a small follow-up study in order to re-look at the lives of the youngest generation in particular and to see how far they had followed their anticipated trajectories. Two family groups and one individual gave five further interviews (including with one new participant, too young to take part in the original interviews). Interview data from both stages of the research is used here; the diaries did not specifically touch on working lives and are therefore not used here. British Sociological Association ethical guidelines were followed and participants have seen the transcripts of their interviews and publications from the first stage of the research.

I took a narrative approach to the interviews and began with “tell me your life story.” During the interviews I bore in mind that I wanted to cover key transition points in people's lives: leaving school and getting a job; getting married; having children; and so on, as well as family history, as far as this was known and followed up on these points if they weren't covered by the participant. A focus on major life events and rites of passage helped to show whether these decisions had a lasting impact or not. Interviewing multiple generations of the same family meant that I could identify what is passed on through the generations, particularly in relation to work biographies. The transcripts were analyzed both thematically and as narratives (Czarniawska, 2004). The biographies used here have been chosen because the stories relate to key issues in patterns of working lives: the idea of a “job for life,” the gendered nature of working lives, family influences and deindustrialisation. The NS-SEC classifications of the participants' jobs/ former jobs range from 1–6, with only the youngest falling into “8 Never Worked” (ONS, 2010). All own their own homes (either with a mortgage or outright). In a qualitative study of this sort it is not feasible to gather a respondent sample who are statistically representative of the place. My sample are skewed toward administrative and intermediate occupations, perhaps partly because more women than men took part. I would argue, though, that although the group may not be statistically typical of the local population they are not atypical either. For the most part, they fall within what Byrne (2019) termed “the missing middle” of those who are reasonably comfortably off and “missing” because they are often not accounted for in sociological class analyses.

Wigan is one of the oldest towns in the historic county of Lancashire, having been granted a Royal Charter in 1246. Coal mining took place in and around the town from at least the 1500s until the 1990s, with the last mine closing in 1992. Mining is an extractive industry, literally tearing the place apart to provide fuel for the industrial revolution and the growth of the British empire. Textiles also became an important industry drawing people to the town in the nineteenth century (Wigan Archaeological Society, 2019). This was already in decline by the 1960s and Wigan therefore did not attract South Asian migrants in the ways that other mill towns around Manchester did. Since the demise of coal and textiles there has been a lack of employment, particularly low-skilled jobs, in the local area. According to Office for National Statistics figures, currently the unemployment rate is below the north west average (3.5 and 4%, respectively), but the percentage of people who are economically inactive due to long term sickness (31.8% of the economically inactive) is higher than the average for the north west (26.1%) (Nomis, 2019). As Lisa Nandy, the local MP has pointed out (Nandy, 2020), recent government policy has been to focus job opportunities on cities rather than towns. This has left Wigan and other places around a large city unable to attract new employers to the locality. There is a strong sense of belonging to place, or what Tomaney and Pike (2020) call “enduring local attachments,” which mean that people stay here. In the 2016 referendum the town voted 63.9% to leave the European Union. This was not discussed in any of the interviews. Wigan today could be seen as a “typical” northern English, deindustrialised town, albeit with less in-migration than some other similar places (Beatty and Fothergill, 2018; Rhodes et al., 2019).

Wigan may be seen as a “left behind” place (Rhodes et al., 2019) by some, but those I spoke to were keen to highlight its benefits describing it as “quite a nice place to live” and pointing out the excellent transport links and proximity to green spaces (Prince, 2014). It does have what Wigan Archaeological Society (2019) describe as an “image problem,” often blamed on Orwell's *The Road to Wigan Pier* describing life there in the 1930s, which can transfer to the people who live there (Allen and Hollingworth, 2013; Prince, 2014; Pedersen and Gram, 2018; Rhodes et al., 2019). There are a number of industrial “relics” which point to its Victorian industrial heyday including mill buildings and the elaborate former Wigan and District Mining and Technical College building now used as the town hall. None of my respondents worked in mining or mills themselves but all had parents or grandparents or other family members who did. The current college has an old winding wheel outside to signify the connection to the mining college (one of the foremost in the country at the end of the nineteenth century). The loss of industry is therefore a constant, but silent, “reminder” in the landscape. Not that the work available in either coal or cotton industries was pleasant, the industrial taskscape was at least cruel and sometimes lethal.

Table 1 details the participants included here and their relationships. All participants have been given a pseudonym. Where I did not speak to the person themselves then they are referred to by their relationship e.g., Janet's husband.

**Table 1:** The participants.

**Grandparents**	**Parents**	**Children**
**“*****The Leythers*****”**		
“Leyther” is a person from Leigh (a separate town within the Metropolitan area of Wigan)	**Claire**, 35, works in recruitment, currently for a Government agency; lived in Leigh all her life	2 daughters, 10, and 3
**Val**, 65, retired bank clerk lived in or close to Leigh all her life **Completed Diary**	**Paul**, 35, married to Claire, spent 5 years out of Leigh in the army, works as a technician	
**Keith**, 66, retired senior manager for international company, lived in Leigh 20 years	**Rob**, 37, lives in London with partner, works in media, brief email “conversation”	1 son, 1 year old
***John and Joanne***		
	**Joanne**, 35, primary school teacher, always lived in Wigan	2 children, 5 and 1
**John**, 61, retired BT engineer, always lived in Wigan **Completed Diary**	Joanne's husband, BT engineer from Wigan	
Wife, teaching assistant, always lived in Wigan	Joanne's sister, 33, works in a health care profession, lives in Wigan, single	
***Barbara***		
**Barbara**, 75, retired coach tour guide, widowed and remarried, always lived in Wigan **Completed Diary**	Daughter, married, works part-time, lives in a neighboring town	3 children 10 and under
**“*****The Aspinalls*****”**		
**Beryl**, 76, married, retired administrator, always lived in Wigan **Completed Diary**	**Janet**, 52, at first interview in 2010, does not work due to a disability, formerly civil servant, lives close to Mum Beryl, husband, not interviewed, insurance assessor, also from Wigan **Completed Diary** Interviewed again in 2016	**Tom**, 18, taking A levels, hoping to study Computing at university **Completed Diary Lauren**, 16, taking GCSEs, wants to be a solicitor **Completed Diary** Both interviewed again in 2016 Tom aged 24 in his first graduate job in IT, Lauren aged 22 just returned from a post-university gap year
**Ian**, 62, Beryl's brother, married, retired teacher, lived in Manchester and Salford when younger	2 children, daughter lives in Thames Valley, son about 35 miles from Wigan	1 baby grandson
***Linda's family***		
**Ethel**, 85, widowed, always lived in Wigan, 5 children, 1 great-great-grandchild	**Linda**, 61, retired secretary, always lived in Wigan, husband an engineer from Scotland originally **Completed Diary**	**Kate**, 34, at first interview in 2010, works at Leisure Center, divorced, 2 children, including **Josh** interviewed in 2017, when he was 18
***Sean***		
Father, deceased, ran own business Mother, widowed, lives close by	**Sean**, 42, lives with partner, always lived in the same house (from 1 year old), sheet metal worker	2 sons, 6, and 2

*Names in **bold** indicate those interviewed. Those who completed diaries are also indicated. All participants are White British*.

The following analysis examines the working lives of those entering the workforce at different times between 1940 and 2017. In looking across whole lives the dynamism of working life comes to the fore: various strands of personal choice, opportunities afforded by the place and time, and family support are woven together to create a cohesive biographical narrative.

## A Job For Life and the Demise of Industry

A “job for life” is something that is understood to belong to an industrial era. With insecure, part time contracts now the norm in many sectors (Standing, 2009; Rhodes et al., 2019), a job for life would appear to be a luxury. Indeed, Atkinson (2010) found that even highly qualified professionals no longer saw themselves as necessarily able to pursue a single career throughout their working life. However, in the industrialized past, it was not unusual (Strangleman, 2015). Rather than a single job what this often meant was working for a single employer and working one's way up.

“*I started work for … company and worked for them for the rest of my working life, it was fine, not in the same place all the time, moving from job to job, factory to factory [all around Wigan/St Helens] … and was working there when I retired [after 40 years].”*

*(Keith, 65, 2009)*

Keith is not a “born and bred” northerner but moved north with his family when he left school at 15 in 1959. He took up an apprenticeship and stayed with the same company until he took early retirement. However, he did not work in the same role throughout and retired as a senior manager for the organization. He told me that he was taught “how to say things in a Northern accent” by a fellow apprentice to help him fit in better, although as a senior manager his comparative lack of a local accent may well have worked to his advantage in conferring a more middle-class identity. This career path was possible because night school offered people (men, in my sample) the opportunity to continue their education whilst being able to work and support a family:

“*I did a fair amount of my further education in St Helens and Wigan so having left school with minimal, well not adequate, qualifications so it really was necessary to go to night school so I did, well I was still going to night school when I was 36, I think.”*

*(Keith, 65, 2009)*

During the 1960s and 1970s this was an accepted career path (Byrne, 2019, p. 96). Janet's husband, in the 1970s, also left school at 15 and took up an apprenticeship but continued to gain qualifications:

“*[he] did leave school, but he didn't stop being educated, he carried on … that doesn't exist anymore, the old apprenticeships, which is what he went through… he must've left school in about 1970, so 15 years later he's still actually in education.”*

*(Janet, 52, 2010)*

This type of career path gave these men the opportunity to experience the workplace without having to cram tertiary education into 3 years in a university context. At 15, neither wanted to stay on at school but in the 1960s and 1970s this did not preclude them from gaining qualifications throughout their working lives and pursuing professional/senior management careers. Whilst their schooling and their first job put them into a “technical occupation” (NS-SEC class 5), their ultimate career destination put them into NS-SEC class 1 (ONS, 2010), demonstrating the “dynamism” of social class that Byrne (2019, p. 4–5) highlights. Keith's son and Janet's children all went on to higher education, continuing the upward absolute social mobility of their parents.

In contrast, after the 1950s and 1960s “golden age of smooth and rapid school-to-work transitions” (Roberts, 2004), Sean left school at sixteen in the early 1980s and also took up an apprenticeship. This in itself was an achievement at that time: “I did well for get that ‘cos there was 800 people going for 60 jobs” (Sean, 42, 2009). However, despite serving his time including going to college for 4 years on day release from work and getting his ONC (a qualification equivalent to “A” levels) he felt “it's not done me any good.” Unlike Keith, Sean has moved to different organizations. There is no particular career trajectory, or aim, simply for work that is challenging and interesting:

“*Right boring job that, when you serve your time it's all right when you're in factory and then when I made … my time I worked three months on a press and I thought when I'm 21 I'm leaving, so I was 21 Wednesday, Friday I left.”*

*(Sean, 42, 2009)*

Although Sean has never experienced any lengthy periods of unemployment he has had to find new jobs due to redundancy and the threat of job loss is ever-present, making him fearful for his children's future:

“*then I ended up going GEC where they make power stations - it's all flat now that … they're not bothered about manufacturing so it's all gone … so now I'm working back in Wigan … but last week there were 15 redundancies … our hours have been cut down to 30 hours … in Wigan there's no employment, back in th'olden days it was all mining and engineering but now it's all gone so when my lads leave school what [are] they going to do?”*

*(Sean, 42, 2009)*

The “olden days” of mining and engineering jobs were as recent as 1950s and 1960s when, as Keith's story shows, people were moving to the area for work and getting an apprenticeship could secure a secure job for the future. The working landscape of the place where Sean's parents and grandfathers (both miners) lived and worked has changed so that there is no “family script” (Bertaux and Thompson, 1996) to follow, but there is a “residual culture” (Williams, 1958; Byrne, 2019) which leads Sean to feel lost between the Wigan he grew up in with industrial work the norm and the ongoing deindustrialisation he is experiencing now. Even those with “traditional” jobs are acutely aware of their precarity (Standing, 2009) and the uncertainty for future generations, particularly for “masculine” work (Walkerdine and Jimenez, 2012).

Along with similar towns overshadowed by nearby cities, Wigan has failed to attract new manufacturing or technical work (Rhodes et al., 2019). Keith confirms Sean's view of the current situation for those who, like Sean, do not see themselves as “academic”:

“*Construction is the only area left for people who would have traditionally gone into the pits or the mills, there really isn't that work available now, it tends to be a McDonalds, or an Asda or things like that that people find themselves going to ‘cos they've not got the academic qualifications.”*

*(Keith, 65, 2009)*

This is the situation the youngest person I spoke to finds himself in. Josh, who was 18 when we met in 2017, was part of the first cohort in England to have to stay in education or training until 18. Unlike his stepsisters who all have degrees and are employed in public sector roles, Josh does not want to go into higher education and wants a more practical career. His uncle and great uncles work in construction, but Josh has been unable to find an apprenticeship. In any case, modern apprenticeships, whilst often being as competitive as Sean's was, do not necessarily lead to a permanent job. Josh has been compelled to go to college to undertake a BTEC (level 3) which he has no interest in: “it's boring” he told me, although he also echoed Roberts (2011, p. 28) participants in saying that he just gets on with it. It is perhaps amongst young men such as Josh that the biggest changes can be seen over the generations as jobs requiring vocational qualifications have all but disappeared (Roberts, 2004). Compared to the experiences of Keith and Janet's husband in the 1960s and 1970s discussed above, opportunities for those who prefer practical to academic work are far fewer now in a place like Wigan (McDowell, 2019). Josh is embedded within a place and family of male relatives who do practical work which clearly demonstrates that his situation is a result of structural changes, rather than personal choices (Byrne, 2019:58–59).

The working landscape of Wigan is such that those with higher educational qualifications may need to move away to find appropriate work, especially outside the type of infrastructural public sector roles, such as teaching and health work, where Josh's step-sisters are employed (Finn, 2017; Pedersen and Gram, 2018). Ian, Janet's uncle described such workers— “policemen, firemen, local authority employees” —as “the aristocracy of the working class.” They lived on a council estate alongside him, growing up in the 1950s. Today they would be more likely to live on private estates of semi-detached 1960s housing, as indeed many of the participants do. This kind of work, much of which now requires a degree, is available and needed everywhere. Rob, Val's son, who lives in London, exemplified this dilemma when he explained that he felt his media work colleagues would find it strange if he chose to live in a place like Leigh (Rob, 37, 2010, via email). As Ian told me in 2010, he could count 24 children of his neighbors who had all been to university but only two still live in the Wigan area “one's a teacher and one's an accountant,” the kind of professions that are needed everywhere.

As Nilsen and Brannen(2014, p. 1.1) point out “lives need to be understood over time and in particular times and places.” Both structural changes to the education system and economic and political changes through globalization, deindustrialization, and neoliberalism have made the working taskscape in Wigan in 2017 very different from what it was in 1959, when Keith started work (Byrne, 2019). It is noticeable here that it is men who have felt the greatest impact of these changes. I will now examine some changes to the work of the female participants.

## Women'S Work: the Present and the Past

In the 1940s and 1950s a young person's wage would have helped to support parents and younger siblings (Nilsen and Brannen, 2014). None of the participants here in either the grandparent or parent generation left home before marriage (or partnership) except to go to university or join the armed forces.

Ethel, in her mid-eighties when we met in 2010, left school at 14 years old during the Second World War. It was easy enough to find a job as a school leaver as she explained:

“*There were two jobs came up, one was in an office, and then this came up [in a bakers], because me dad knew the man who delivered the yeast for this shop and he told me dad that they were wanting an apprentice you know so I went there…and I worked from starting school to getting married, just the one place.”*

*(Ethel, 83, 2010)*

Ethel took the job that sounded most attractive to her without any specific long-term plans but she has used the skills she learned throughout her life. Ethel also points out that she stayed at the bakery until she married, and how happy she was there, being treated as one of the family. Barbara on the other hand was “pushed” into secretarial work as her father paid for a shorthand and typing course after leaving school. She made use of strong social ties through going to work at the factory where both her father and, earlier, her grandfather had worked (Byrne, 2019). Although she worked at other places too, she returned there after her daughter was born.

“*…me dad was a joiner by trade but he, … I mean he was a works manager, but that's how he started off with an apprenticeship… it was at Whitter's at Appley Bridge, which was a linoleum factory, and I went there … so there was three generations of us who had gone to the same firm… I went to Upholland Grammar School, left there [at 16] … went to a commercial college for 3 months, did shorthand typing and bookkeeping and then started in the office down there and [after birth of daughter] I was back at Whitters so I was still working locally.”*

*(Barbara, 73, 2010)*

Barbara's path, although her father was “works manager,” followed a typical meandering “women's work” route, including these elements of circularity, leaving and then returning to the same place (without the career progression of her father). But Barbara also emphasizes the “family script” (Bertaux and Thompson, 1996) that she followed in getting a job at the local factory alongside her father. Although Barbara attended a grammar school her career showed little difference from those who had left elementary school at fourteen. Beryl, like Ethel, left elementary school at fourteen. She also undertook a shorthand and typing course paid for by her father. As with Barbara, this gave her access to a variety of jobs both before and after marriage. Once working the young women would have contributed significantly to the household budgets; there was no question of them leaving home until marriage (Bennett, 2015).

Val and Janet could be said to have had a “job for life,” but had no significant progression in their careers as their husbands (above) did:

“*I worked in a bank from being 16 … you didn't have maternity leave then, you got pregnant you lost your job, but I did go back to the same branch part-time after, when [Rob] started school and [Claire] was three.”*

*(Val, 63, 2009)*

“*I decided to leave [school] at eighteen and it was 1976, I thought … go out and get a job, which you could do in those days, and I kept that job, that was the only job I ever got….I worked for the Civil Service and in the same office doing the same job until I was thirty eight, nine when … I retired on ill health….there was maternity leave then [1991], but not as much* ***-*** *12 paid weeks and then half. I [took] eight months in total … then I went back part-time after he was born which was, sort of, available - you only had to make your own case…”*

*(Janet, 52, 2010)*

Val was the first in her family to go to grammar school, leaving at 16 in the early 1960s, despite school staff encouraging her to stay on and consider a teaching career. Janet was offered a place at grammar school but chose to attend a comprehensive, as grammar schools were about to be abolished in Wigan. Despite having “A” levels and the option of going to university (for Janet), or staying on at school and applying to teacher training college (for Val), both women followed the example of the previous generation and fitted their work around family and children (Bennett, 2015), perhaps embodying the circularity that lives, over time, tend to follow (Brannen, 2006). In contrast men of the same generation who went to grammar school—Ian, Janet's uncle, is an example here—did go on to university in the 1960s, and this seemed to be the accepted path amongst his cohort. Although the career paths of women have changed in many ways since the mid-twentieth century, there are also similarities in the way women view their working and family lives today.

The ability of both Val and Janet to retain or regain work in organizations where they were known is an example of the “industrial citizenship” and the family orientation of many organizations in the past (Standing, 2009; Strangleman, 2015). Since 1996 employees in England have had the right to request flexible working but these examples from the 1970s and early 1990s show that larger employers and the public sector were open to requests before it became a statutory requirement (Crompton, 2006, p. 106). Val's parents and in-laws helped with childcare. Janet paid for childcare as her mother was still working (Brannen, 2003). The dual-earner/female-part-time-carer combination became normative from the 1970s onwards, but, according to Crompton (2006, p. 196), has not resulted in any significant change in gender roles. It is perhaps notable here that despite lengthy careers neither Val nor Janet was promoted, in the way Keith (above), for example, was.

Claire (Val's daughter) changed her career path to fit around family when she had her second child, an example of how different life stages can trigger changes: “I used to be the money earner but then I took a backstep to work here. Though it's a good job, I reduced my earnings.” In moving from the private to the public sector Claire now has job security and greater flexibility albeit with a cut in salary (Crompton, 2006, p. 106). Gerson (2010), in the “The Unfinished Revolution” shows how young women in the US accept that they need to take responsibility for supporting themselves and their children without relying on the children's fathers. Claire and Joanne are not single parents but do shoulder the greater responsibility.

Joanne has, to a greater extent, taken a more active career path as a primary school teacher and is the primary earner in her household:

“*I'd love to [go part time] but … my job is so secure and I have gradually worked my way up in school and I love my job … I'm very well paid so you know it helps us to manage. Like I say, [husband] was made redundant just as we moved here and then got this job, so we never rely on it, you know … it's important to rely on me…”*

*(Joanne,35, 2009)*

Joanne had a career path in mind when she left school as she studied Primary Education at a local university. The job itself “came up” when she was finishing her degree, was local (she was living with her parents at that time) and she has been there ever since.

She sees domestic duties as being her responsibility, regardless of whether she carries them out or not:

“*… [husband] has two days off in the week and he can apply for overtime or he does my cleaning for me which is great! I said that was my condition, if I go back to work I need help and he is very, very good, he does an awful lot … in the house, you know, round about, and at weekends you know, he'll, er, take [son] out and about.”*

*(Joanne,35, 2009)*

Both these women rely on grandparents for at least some childcare, as is common (Brannen, 2006, p. 133). For this reason, neither would consider moving away from Wigan. Both have higher qualifications than their husbands, neither of whom has a degree. The continuation of women becoming entangled in domestic work either in addition to or to the detriment of paid work outside the home is an ongoing issue (Gerson, 2010; Charbonneau et al., 2019). Crompton (2006, p. 141) points out that men with higher levels of education do more housework in general, but Joanne is not unusual in continuing to take responsibility for the care of the home and children (Lyonette and Crompton, 2014; Charbonneau et al., 2019). The fact that Joanne's husband is at home during the week and uses this time to do housework implies they are at least partially following the “time availability hypothesis,” in terms of time spent on housework, but also continuing to “do gender” in that Joanne assumes it is still her responsibility (Lyonette and Crompton, 2014). With both these couples the men do a more equal share of childcare than of housework tasks, which again is in line with findings elsewhere (Lyonette and Crompton, 2014). It may be that socially conservative places, such as Wigan (Nandy, 2020), are slower to change in relation to attitudes to gender. This slower pace of change was mentioned by Keith in his interview in 2009 in relation to attitudes of his charity work colleagues toward domestic violence and by a council officer I spoke to as part of this research project in relation to local attitudes toward disability and sexuality, and can be understood as the legacy of a strongly patriarchal culture in the industrial past (Bennett, 2015). Overall Claire and Joanne have been able to continue with more challenging careers than the previous generation of women, but they are still left with the main responsibility for the housework and children (Brannen, 2003; Gerson, 2010).

Systemic changes in the working landscape of Wigan are different for women with families than for men undertaking more manual roles. “Women's work” is changing more slowly being driven by broader changes in gender structures in society, rather than substantial changes to the types of work available.

## Discussion and Conclusion

By focusing on a whole work-life biography a cohesive view of how work is negotiated and juggled alongside “life” has been explored here. The social practices of working life have changed considerably for men, in Wigan, over the last 80 years, perhaps less so for women. Those whose education took place after the 1944 Education Act seemed to see their lives as successful and fulfilling. Since the 1970s however, opportunities for work and for education have contracted. Degree-equivalent education was available to many prior to the recent expansion, as Keith demonstrates, without the costs involved today. Women continue to juggle paid work and family or care work, made more difficult by the need to secure a full-time income. The influence of family members and other social networks has decreased along with job security.

It is clear from these biographies that that whilst there have been changes over the last 80 years in the way working life biographies develop through the lifecourse, there are a number of continuities as well, once one looks across a longer period. Looking at a life embedded within a taskscape presents a holistic view of the possibilities and threads involved in work-life biographies (Ingold, 2000; Hörschelmann, 2011; Bennett, 2015).

Overall what comes through most clearly from these stories is the contingencies in any working career. The majority of the narratives take winding paths without any clear goal in sight, looking for somewhere to fit in rather than the next promotion opportunity. Although the research is focused on families who have remained in place over multiple generations, it demonstrates the dynamic nature of these lives (Hörschelmann, 2011; Byrne, 2019).

As life transitions become increasingly destandardised and individualized in post-industrialized societies life course research needs to have the focus of this paper on both micro-social agency through individual voices and structural changes and continuities in communities. The lives of women, in particular, are still being shaped by gendered norms, although this is now being ascribed to agency (“I've worked hard … I love my job” from Joanne), whereas previously it was more clearly structural, echoed in Barbara's claim that “mothers didn't work.” This illusion of choice is an aspect of the neoliberal agenda to ascribe agency where it does not properly exist and consequently remove obligations for state support (Standing, 2009).

The taskscape here provides for everyday needs, but not much more. The kinds of work the participants here describe tend to be related to the rarely noticed infrastructure of modern life (Star, 1999). These essential jobs often fade into the background: retail work and banking, teaching, public sector work. Those who want to work in the media (Rob, Val's son), in politics, in research (Lauren), in cutting edge technology, need to look to cities for employment (Nandy, 2020). This perhaps opens up other career options, but it also seems to confirm the commodification of life and the subservience of social and family life to work life. Due to the underlying similarity between most of the jobs undertaken by the participants here, and their common place of residence and family histories, they can be seen to share a taskscape: working (metaphorically) together to reproduce the culture of Wigan (Byrne, 2019). Whether they are described as “the aristocracy of the working class,” lower-middle class or traditional working class, their cultural identity is as a Wiganer (or Leyther). I would argue that place is the determinate identity for these people rather than as someone of a particular social class. Reciprocal recognition is as a Wiganer and a Northerner (with the right accent) over and above being working or middle class. It could be claimed that place trumps class where place attachment and shared working culture and history are strong.

The examples of working lives discussed here have shown how structural changes mean the current generation of women are more able to pursue careers than their mothers but continuities in gender norms still allocate them primary responsibility for family. Deindustrialisation means manual work has almost disappeared and jobs are no longer “for life.” The “missing middle” (Byrne, 2019) in Wigan have working life biographies that have been subject to changes throughout the twentieth century and into the twenty-first. Examining family biographies has demonstrated the dynamic nature of these changes.

## Data Availability Statement

The datasets generated for this study are available on request to the corresponding author.

## Ethics Statement

The studies involving human participants were reviewed and approved by University of Manchester. The patients/participants provided their written informed consent to participate in this study.

## Author Contributions

The author confirms being the sole contributor of this work and has approved it for publication.

## Conflict of Interest

The author declares that the research was conducted in the absence of any commercial or financial relationships that could be construed as a potential conflict of interest.
